# Coexistence through mutualist‐dependent reversal of competitive hierarchies

**DOI:** 10.1002/ece3.3689

**Published:** 2017-12-21

**Authors:** Mohsen Mehrparvar, Sharon E. Zytynska, Adalbert Balog, Wolfgang W. Weisser

**Affiliations:** ^1^ Terrestrial Ecology Research Group Department of Ecology and Ecosystem Management Centre for Food and Life Sciences Weihenstephan Technical University of Munich Freising Germany; ^2^ Department of Horticulture Faculty of Technical and Human Science Sapientia Hungarian University of Transylvania Tirgu‐Mures Romania; ^3^ Present address: Department of Biodiversity Institute of Science and High Technology and Environmental Sciences Graduate University of Advanced Technology Kerman Iran

**Keywords:** aphid, community structure, competition, interspecific interactions, mutualism

## Abstract

Mechanisms that allow for the coexistence of two competing species that share a trophic level can be broadly divided into those that prevent competitive exclusion of one species within a local area, and those that allow for coexistence only at a regional level. While the presence of aphid‐tending ants can change the distribution of aphids among host plants, the role of mutualistic ants has not been fully explored to understand coexistence of multiple aphid species in a community. The tansy plant (*Tanacetum vulgare*) hosts three common and specialized aphid species, with only one being tended by ants. Often, these aphids species will not coexist on the same plant but will coexist across multiple plant hosts in a field. In this study, we aim to understand how interactions with mutualistic ants and predators affect the coexistence of multiple species of aphid herbivores on tansy. We show that the presence of ants drives community assembly at the level of individual plant, that is, the local community, by favoring one ant‐tended species, *Metopeurum fuscoviride*, while preying on the untended *Macrosiphoniella tanacetaria* and, to a lesser extent, *Uroleucon tanaceti*. Competitive hierarchies without ants were very different from those with ants. At the regional level, multiple tansy plants provide a habitat across which all aphid species can coexist at the larger spatial scale, while being competitively excluded at the local scale. In this case, ant mutualist‐dependent reversal of the competitive hierarchy can drive community dynamics in a plant–aphid system.

## 1 INTRODUCTION

The coexistence of similar species in an ecological community has long been studied (May, 1973). Research supports the theory that species must sufficiently differ in their ecology to enable coexistence (Chesson, 2000). Mechanisms that allow for the coexistence of two competing species that share a trophic level can be broadly divided into those that prevent competitive exclusion of one species within a single patch, and those that allow for coexistence only at a regional level, that is, among patches. Prevention of competitive exclusion at the regional level implies that competitive exclusion may occur within each local community patch, but at any one time different patches are occupied by different communities, leading to regional coexistence (Atkinson & Shorrocks, 1981). At the local level, coexistence of two species that share a trophic level is made possible by a range of mechanisms. This includes differential resource uptake, that is, differences in traits of the individual species (Debout, Dalecky, Ngomi, & McKey, 2009), and frequency‐dependent attack by natural enemies, that is, interactions with a third species (Abrams, 1999; Gliwicz & Wrzosek, 2008; Paine, 1966; Slobodkin, 1964; Vandermeer & Pascual, 2006). Predator‐mediated coexistence has been described for a number of systems, from intertidal through to invertebrate terrestrial communities (Belsky, 1992; Berendse, 1985; Connell, 1961; Huntly, 1991; Olff & Ritchie, 1998; Paine, 1966).

Competition between phloem‐feeding insects, such as aphids, can also be driven by interactions with their mutualistic ants. Many aphids are mutualistically tended by ants, which feed on the aphid honeydew and, in return, protect the aphids from natural enemies (e.g., pathogens and parasitoid wasps; Renault, Buffa, & Delfino, 2005; Stadler & Dixon, 2008; Sudd, 1987). Ant attendance can have an effect on the successful colonization of a plant by aphids and subsequent extinction of aphid populations (Addicott, 1978b; Banks, 1962; Senft, Weisser, & Zytynska, 2017; Tilles & Wood, 1982; Wimp & Whitham, 2001). However, the relationship between ants and aphids is not always mutualistic; in some cases, ants prey upon aphids (Billick, Hammer, Reithel, & Abbot, 2007; Sakata, 1994, 1995; Singh, Zytynska, Hanna, & Weisser, 2016; Sudd, 1987). Mutualistic aphid–ant interactions may thus have a crucial impact on the community dynamics and local or regional coexistence of aphids that feed on the same host plant (Addicott, 1978a,b).

The tansy–aphid system is ideal for studying the effect of mutualists on coexistence of aphids. Tansy plants (*Tanacetum vulgare*) commonly host three species of specialized aphid that vary in where they feed on the host plant (top shoots or lower leaves), and in their interaction with ants (Mehrparvar, Mansouri, & Weisser, 2014). Two of these aphid species (*Metopeurum fuscoviride* and *Macrosiphoniella tanacetaria*) both feed on the upper shoots of the plant and are rarely observed coexisting on the same host plant, yet both can be present on different host plants in the same field (Loxdale et al., 2011; Weisser & Harri, 2005). This suggests competitive exclusion at the local scale (individual plant), with coexistence only possible across multiple plants at the field or regional scale. Further, *M. tanacetaria* aphids are not ant‐tended, whereas *M. fuscoviride* are highly dependent on their mutualistic interaction with ants (Senft et al., 2017; Weisser, 2000). *Metopeurum fuscoviride* aphid colonies visited more frequently by *Lasius niger* ants had higher colonization success at the beginning of the season, and those visited more frequently by *Myrmica rubra* had lower colonization success, lower persistence, and higher extinction rates (Senft et al., 2017). A third aphid species, *Uroleucon tanaceti*, feeds on the lower leaves of the plant, is not ant‐tended, and is found coexisting locally with both the other aphid species in the field. It is toxic to predators, which could mediate predation pressures for the other coexisting aphid species in the community (Mehrparvar, Mahdavi Arab, & Weisser, 2013). Understanding the outcome of aphid–ant interactions within these local communities can help us to understand the processes driving local competitive exclusion, but regional coexistence of species.

In this study, we used both greenhouse (highly controlled community composition) and field experiments (natural colonization of ants and other aphid predators) to understand the relative effect of competition, predation, and mutualism on the coexistence of different species of aphid herbivores on tansy plants. In particular, we addressed the following questions: (i) What is the effect of ants on the individual occurrence and abundance of the three aphid species? (ii) How do ants affect the outcome of competitive interactions among the three aphid species? and (iii) What are the consequences of ant presence on the coexistence of the three aphid species?

## 2 MATERIALS AND METHODS

### 2.1 Study system

Tansy (*Tanacetum vulgare*) is a perennial herbaceous composite from Europe and Asia (Mitich, 1992) which preferentially grows in disturbed, well‐drained, and poor soils. It often forms as isolated patches alongside river valleys, railway tracks, and on abandoned areas in cities or brown fields. Single plants comprise a “genetically identical” genet with up to 100 flowering ramets (shoots), but usually much fewer. In Jena, Germany, eight aphid species have been found on tansy (Mehrparvar, personal observation) of which the three specialist species, that is, *Macrosiphoniella tanacetaria* (Kaltenbach), *Metopeurum fuscoviride* (Stroyan), and *Uroleucon tanaceti* (L.) (Aphididae) are the most common. *Macrosiphoniella tanacetaria* is not ant‐attended and feeds in loose colonies mainly on the top of shoots. *Metopeurum fuscoviride* is an obligatory myrmecophilous aphid which is commonly attended by the black garden ant, *Lasius niger* (Benedek et al., 2015; Flatt & Weisser, 2000; Mehrparvar, Zytynska, & Weisser, 2013; Mehrparvar et al., 2014), but also by other species such as the common red ant, *Myrmica rubra* (L.) (Senft et al., 2017). *Metopeurum fuscoviride* feeds in more compact colonies near the apex of ramets but can also occupy (at least to a certain extent) the same feeding niche as *M. tanacetaria*. Mixed colonies are very rarely observed in the field (Loxdale et al., 2011). Reduced survival and reproduction of *M. fuscoviride* have been shown when aphids are not ant‐tended (Flatt & Weisser, 2000). The third species, *U. tanaceti*, feeds on the underside of lower leaves of its host plant and is also not ant‐tended. Loxdale et al. (2011) and Mehrparvar, Zytynska et al. (2013) discuss the life cycle of *M. tanacetaria* and *M. fuscoviride*. Both species are monoecious and holocyclic on tansy, but whereas the males of the former species are winged, those of the latter are wingless (Blackman & Eastop, 2006). A wide range of natural enemies such as parasitoid wasps, ladybirds, lacewings, syrphid flies, predatory bugs, and spiders attacks these aphids. Even though the aphid species occupy different parts of a host plant, there may still be indirect competition for phloem nutrients (e.g., Moran & Whitham, 1990).

The experimental system, comprising the tansy plants, the three specialized aphid species, and two ant species (*Lasius niger* and *Myrmica rubra*), was studied using a greenhouse and two field experiments performed in Jena, Germany, at the Jena Experiment site on the northern outskirts of the city (50.95^°^N, 11.63^°^E) and the botanical garden of Jena (50.93^°^N, 11.58^°^E).

#### 2.1.1 Greenhouse experiment

The aim of this experiment was to determine the interactions among the three aphid species on tansy in the presence or absence of ants and absence of other potential natural enemies.

##### Experimental plants and insects

Tansy rhizoms were collected from multiple plants near the Institute of Ecology in Jena and were planted in three‐liter capacity pots filled with soil in May 2009. Plants were maintained outside until the developed shoots had reached a height of 20 cm. Plants were then transferred to the greenhouse (~25°C during the day and ~20°C at night and with a 16‐hr L: 8‐hr D light regime). Adult individuals (apterous viviparous females) of the three aphid species were collected from multiple colonies and plants from the field and transferred to the greenhouse for the experiment. Colonies of the two ant species, *L. niger* and *M. rubra*, were collected from the same field as the tansy stolons and were thereafter maintained in the greenhouse. Each ant colony had several hundred workers, many ant larvae, and pupae. The colonies were housed in 10‐L volume buckets filled with humid soil, the inside of which was coated with Fluon (Fluoropolymer Dispersion, Whitford GmbH, Germany), which acts as an ant barrier keeping them from escaping. The ants were offered boiled egg as protein in addition to carbohydrate resources that they would obtain from the aphids. The soil in the buckets was sprayed frequently with water to avoid effects of desiccation.

##### Experimental design

This experiment was undertaken in the greenhouse, in the absence of natural enemies, and using a fully factorial randomized block design with 10 blocks. The main experimental treatments were three ant treatments (with *L. niger*, with *M. rubra* and without ants), each one with seven aphid treatments. The aphid treatments included each aphid species on its own, each aphid species with one another aphid species, and all three aphid species together, that is, *M. tanacetaria* alone; *M. fuscoviride* alone; *U. tanaceti* alone; *M. tanacetaria* + *M. fuscoviride*;* M. tanacetaria* + *U. tanaceti*;* M. fuscoviride *+ *U. tanaceti*; and *M. tanacetaria* + *M. fuscoviride* + *U. tanaceti*. In total, there were 3 × 7 = 21 treatment combinations with 10 replicates (one replicate per treatment in each block), totaling 210 plants.

Two adult individuals of each aphid species were placed onto each given experimental plant treatment using an additive design, such that when there were two or three aphid species together there were still two adults of each aphid species on the plant. After about 4 hr, subsequent to the plant infestation by aphids, the worker ants were allowed access to the aphid infested plants of the ant‐tended treatments. For each block, two buckets containing the colonies of the two ant species were placed in the experimental arena, that is, each ant colony attended all appropriate aphid treatments within a block. Access of the appropriate ant species was regulated by a series of bamboos sticks (~5 mm diam.). These connected the buckets housing the ant colonies to the plants. Plant pots were placed into water‐filled plates to prevent the escape of worker ants as well as the access of vagrant workers in the greenhouse to no ant‐treatments. The plants in non‐ant treatments were not connected to any ant colony. All plants were irrigated gently every time they required water.

To obtain cohorts, adult aphids were allowed to reproduce for 48 hours whereupon the numbers of adults (0, 1, 2) were counted to assess early adult survival in the treatments. Then the adults and all offspring except three 1st instar nymphs were removed from the plants to obtain populations of similar reproductive age. When no initial adults survived, 1st instar nymphs from stock colony plants were added. Following this, the number of aphids was counted each day for 20 days.

The variables used for analyses were as follows: The number of adults initially put on the plant that survived until the second day (*early adult survival*); the *cumulative number of individuals* (the sum of all daily aphid counts, as measure for population growth and productivity for 20 days); and, *colony persistence*, calculated as the number of days until no aphid was present any more on the plant, up to day 20.

#### 2.1.2 Field experiments

The aim of this experiment was to determine the competition between aphid species and to assess the colonization success of aphids on tansy in the presence or absence of ants in the field when natural enemies are present.

##### Competition experiment

Here tansy rhizoms were collected, cultivated, and maintained as in the greenhouse experiment, and we used the three tansy aphid species as before. In August 2010, colonies of *L. niger* were located within an experiment grassland site for use in the experiment.

*Experimental design:* The experiment involved a randomized block design with 30 blocks. Within each block, plants of approximately the same height and number of leaves were used. There were two ant treatments, that is, with and without access of workers of *L. niger*, and seven combinations of aphids (same as in the greenhouse experiment), resulting in total 2 × 7 = 14 treatment combinations, and replicated 30 times with one replicate per block. The 14 plants of a block were placed around one ant colony with a distance of 1 m between pots. Before placing the plant pots in the field, all small plants around the place where a plot was placed were re‐moved at ground level, to exclude the access of ants to the experimental plant via the aerial parts of nearby plants. For “without ant” treatments, each potted tansy plant was placed in another empty pot without holes in its base. Insect glue (Raupenleim grün, Schacht, Germany) was daubed on the outside surface of the pots. The plants were irrigated gently, so as not to disturb the vegetation or aphid colonies, as required. To avoid any build‐up of water within plots, these were checked for daily, but this only occurred once after heavy rain.

The experiment was started by placing two unwinged adult female aphids and five 3rd or 4th instar nymphs, hence a total of seven aphids, on the plant, for each of the three aphid species per experimental plant treatment. After 1 day of the experiment, aphids were checked and if the total number of aphids per species on each plant was less than five, numbers were suitably increased with new individuals. Thereafter, the numbers of aphids were counted each day in the morning for 21 days.

The following variables were used for analysis: the *cumulative number of individuals* after 21 days, that is, the sum of all daily aphid counts, as measure for population growth and productivity; and *colony persistence*, calculated as the number of days until no aphid was present any more on the plant, up to day 21.

##### Colonization experiment

In May 2011, 30 potted tansy plants (~25 cm height) were allocated to two treatment groups, that is, with ant and without ant, with 15 replicates for each treatment. The plants were placed in three rows of 10 plants each with a distance of 1 m between pots. Plants were assigned alternately to ant treatments, such that no adjacent plants were of the same treatment. Before placing the plant pots in the field, all plants were mown at ca. 3 cm height near the places where plots were placed, to exclude the access of ants to the experimental plants. For the without ant treatment, each potted tansy plant was placed in another empty pot without holes in its base, and insect glue was daubed on the outside surface of the pots, as described above. This experiment was performed in the botanical garden of Jena, Germany, where some other tansy plants grow and are naturally infested by aphids over the season. The plants were allowed to be naturally colonized by aphids, and then they checked every week from 23 May until 3 October. The number of winged (adult) aphids and the number of unwinged adult aphids plus nymphs were counted separately.

### 2.2 Statistical analysis

For the greenhouse experiment, generalized linear models using binomial distribution with logit link were used to analyze early adult survival of each aphid species. All analyses were conducted using SPSS version 22.

For both greenhouse and field experiments, the cumulative numbers of individuals at the end of the experiment were transformed as (*x* + 1). Generalized linear models using a Gamma distribution with log link function were used for the analysis. For each aphid species, models included main effects for the competition (aphid combination) and ant treatments, as well as interactions. Aphid competition treatments included the focal aphid species on its own, the combination of the focal with either of the two other aphid species combinations, and the three‐species combination (4 levels). The ant treatment had three levels in the case of the greenhouse experiment (*L. niger*,* M. rubra*, no ant), and two levels in the case of the field experiment (with *L. niger* and no ant).

To analyze colony persistence of the greenhouse and field experiments, survival analysis (Kaplan–Meier) was employed. If the colony survived until the ends of experiment, then the time‐point 20 (greenhouse experiment) or 21 (field experiment) was entered in the survival analysis as censored data. For each ant treatment, a separate analysis was performed using the *log‐rank* test (a test for comparing the equality of survival distributions where all time points are weighted equally) to compare colony persistence of each aphid species pairwise between different competition treatments. Pairwise comparisons between overall effects of each ant treatment on colony persistence of each aphid species were also performed using the *log‐rank* test.

## 3 RESULTS

### 3.1 Greenhouse experiment

#### 3.1.1 *Macrosiphoniella tanacetaria* (untended‐tended)

##### Early adult survival

**Figure 1:**
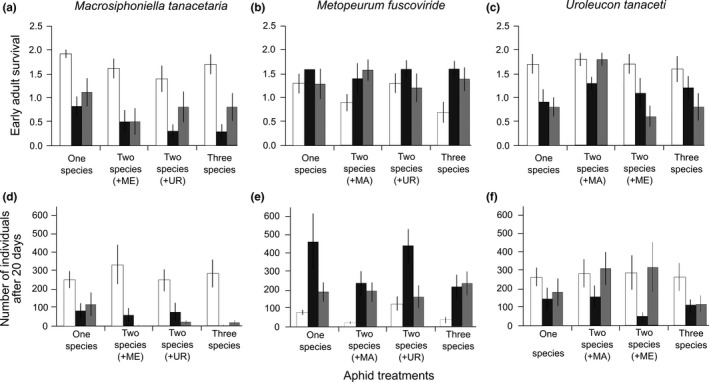
The effect of ant presence and aphid‐aphid competition on early adult survival and population growth (cumulative number of individuals) of specialized tansy aphids in the greenhouse experiment. Aphid competition treatments included the focal aphid species alone, the combination of the focal with either of the two other aphid species, and the three‐species combination. The ant treatment had three levels (*L. niger*,* M. rubra*, and without ant). The graph gives mean (±*SE*) numbers of *Macrosiphoniella tanacetaria* (a and d), *Metopeurum fuscoviride* (b and e), and *Uroleucon tanaceti* (c and f) early adult survival (first 2 days after experiment were started, a–c), and cumulative numbers of individuals after 20 experimental days (d–f). White columns: no ant, gray columns: *Myrmica rubra,* black columns: *Lasius niger*. MA:* M. tanacetaria*; ME:* M. fuscoviride*; UR:* U. tanaceti*

The presence of ants strongly reduced early adult survival; in many cases no adults, and on average fewer than one adult survived until day two, while in the absence of ants, both individuals almost always survived (Figure 1a, Table 1). Workers of both *L. niger* and *M. rubra* were often observed killing and carrying *M. tanacetaria* to their nest (Figure S1). Competition also negatively affected early adult survival, so that the average number of adults when they were accompanied by other aphid species was smaller (Figure 1a). The interaction between the competition and ant treatments was not significant (Table 1).

**Table 1:** The effect of ant presence and aphid‐aphid competition on early adult survival and population growth of specialized tansy aphids in the greenhouse and field experiments

Source	Ant		Competition		Ant × Competition	
	*df*	χ^2^	*df*	χ^2^	*df*	χ^2^
Greenhouse						
Early adult survival						
*M. tanacetaria*	2	43.96***	3	8.36*	6	3.65
*M. fuscoviride*	2	11.10**	3	1.13	6	6.99
*U. tanaceti*	2	18.77***	3	9.15*	6	7.49
Cumulative number of individuals						
*M. tanacetaria*	2	147.37***	3	42.21***	6	75.63***
*M. fuscoviride*	2	72.97***	3	11.02*	6	13.43*
*U. tanaceti*	2	17.35***	3	3.70	6	10.87
Field						
Cumulative number of individuals						
*M. tanacetaria*	1	100.19***	3	23.09***	3	16.55**
*M. fuscoviride*	1	732.97***	3	1.48	3	0.82
*U. tanaceti*	1	5.22*	3	9.14*	3	7.84*

The ant treatment had three levels in the case of the greenhouse experiment (*L. niger*,* M. rubra*, without ant), and two levels in the case of the field experiment (with/without *L. niger*). For the analysis of early adult survival in the greenhouse, generalized linear models with a binomial error distribution and logit link were used. The cumulative numbers of individuals at the end of the experiments were transformed as (*x* + 1) and analyzed using generalized linear models with a Gamma error distribution and log link function.

Significant results are indicated by *p <* *.05, **p <* *.01, ***p <* *.001.

##### Colony persistence and population growth

**Figure 2:**
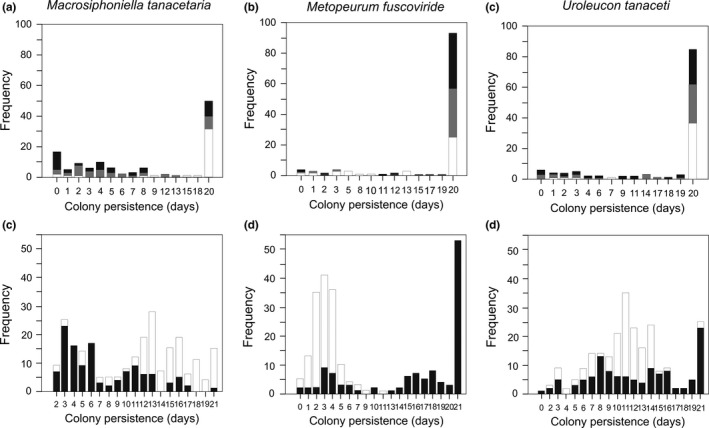
Frequency distribution of colony persistence times (in days) for *Macrosiphoniella tanacetaria* (a and d), *Metopeurum fuscoviride* (b and e), and *Uroleucon tanaceti* (c and F) colonies in the greenhouse experiment (a–c) and field experiment (d–f). The experiment ended after 20 days, and the field experiment after 21 days, and any colony still present in the last day was assigned a persistence time of 20 (21) days. Each graph summarizes all replicates for a focal species across all ant treatments. The ant treatment had three levels in the case of the greenhouse experiment (*L. niger*,* M. rubra*, and without ant), and two levels in the case of the field experiment (with/without *L. niger*). White area of bar: no ant, gray area: *Myrmica rubra,* and black area: *Lasius niger*

Both the presence of *L. niger* and *M. rubra* caused a drastic reduction in colony persistence of *M. tanacetaria*, whereas in the absence of ants on the plant, colony persistence was on average about 2.5 times as long (Figure 2a and Table 3). Competition had no significant effect on colony persistence in the no ant treatment (Table 2). In the presence of the ant *L. niger,* the shortest colony persistence was observed when all the three aphid species were on the plant, while it was four times longer when *M. tanacetaria* was alone (Table 2). In the presence of the ant *M. rubra*, the longest colony persistence was also in the “no‐competition” treatment, while the presence of *M. fuscoviride* on the plant caused a significant decrease in colony persistence (Table 2).

**Table 2:** The effect of the presence of ants and aphid‐aphid competition on colony persistence, in days, of three specialized tansy aphids in the greenhouse and field experiments

	One species	Two species			Three species
		(+MA)	(+ME)	(+UR)	
Greenhouse					
*M. tanacetaria*					
Without ants	19.80 ± 0.20^a^†		15.10 ± 2.59^a^†	18.00 ± 2.00^a^†	16.40 ± 2.11^a^†
*Lasius niger*	10.70 ± 2.68^a^‡		6.90 ± 2.90^ac^‡	8.10 ± 2.72^ad^‡	2.60 ± 0.76^bcd^‡
*Myrmica rubra*	11.50 ± 2.46^a^‡		3.60 ± 0.62^bd^‡	9.90 ± 2.60^ae^‡	4.50 ± 1.84^cde^‡
*M. fuscoviride*					
Without ants	20.00 ± 0.00^a^†	10.60 ± 2.41^b^‡		15.90 ± 2.28^ab^†	11.80 ± 2.87^b^‡
*Lasius niger*	18.20 ± 1.80^a^†	17.10 ± 2.10^a^†		20.00 ± 0.00^a^†	19.20 ± 0.80^a^†
*Myrmica rubra*	17.30 ± 1.98^a^†	17.90 ± 1.99^a^†		17.40 ± 1.80^a^†	18.30 ± 1.70^a^†
*U. tanaceti*					
Without ants	18.30 ± 1.70^a^†	20.00 ± 0.00^a^†	16.90 ± 2.10^a^†		18.10 ± 1.90^a^†
*Lasius niger*	15.20 ± 2.61^ab^†	14.90 ± 2.55^ab^‡	10.50 ± 2.54^a^‡		17.10 ± 2.10^b^†
*Myrmica rubra*	12.90 ± 2.92^a^†	18.00 ± 1.46^a^†‡	16.80 ± 1.92^a^†		12.10 ± 3.05^a^†
Field					
*M. tanacetaria*					
Without ants	16.53 ± 0.66^a^†		16.17 ± 0.67^a^†	12.57 ± 0.78^b^†	11.57 ± 0.75^b^†
With ants	11.10 ± 0.52^a^‡		5.70 ± 0.74^b^‡	6.37 ± 0.58^b^‡	5.70 ± 0.9^b^‡
*M. fuscoviride*					
Without ants	3.07 ± 0.30^a^‡	2.90 ± 0.25^a^‡		3.30 ± 0.27^a^‡	2.87 ± 0.35^a^‡
With ants	15.37 ± 1.32^a^†	15.60 ± 1.38^a^†		16.40 ± 1.13^a^†	14.03 ± 1.42^a^†
*U. tanaceti*					
Without ants	11.23 ± 0.46^a^†	10.97 ± 0.81^a^†	10.80 ± 0.29^a^‡		9.97 ± 0.65^a^‡
With ants	10.10 ± 0.84^a^†	9.30 ± 0.73^a^†	16.37 ± 0.88^b^†		15.13 ± 1.10^b^†

The ant treatment had three levels in the case of the greenhouse experiment (*L. niger*,* M. rubra*, without ant), and two levels in the case of the field experiment (with/without *L. niger*). For the analysis of colony persistence, a survival analysis (Kaplan–Meier) was used. Pairwise comparison between treatments was performed using the *log‐rank* test. Mean (±*SE*) of colony persistence in different aphid (competition) treatments in the presence and absence of ants during the 20 days of greenhouse experiment and 21 days of field experiment is given. Means in rows with different letters and in columns with different symbols are significantly different from one another (*p *<* *.05).

Considering cumulative number of individuals, the interaction between ant and competition treatments was significant (Table 1). The cumulative number of individuals of *M. tanacetaria* after 20 days was more than 12‐fold, and about eightfold, in the no ant treatment than in *M. rubra* and *L. niger* treatments, respectively (Figure 1d, Table 1). Competition reduced the cumulative number of *M. tanacetaria* individuals (Table 1), so that the number decreased to one‐third, half, and one‐fifth in the presence of *M. fuscoviride*,* U. tanaceti,* and in the combination of all the three species, respectively.

#### 3.1.2 *Metopeurum fuscoviride* (ant‐tended)

##### Early adult survival

In the presence of ants, early adult survival was generally better; in most cases both adults survived. In the absence of ants, on average only about 0.5 adults survived in the first 2 days (Figure 1b, Table 1). Competition had not significant effect on early adult survival (Table 1). There was also no significant interaction between the ant and competition treatments (Table 1).

##### Colony persistence and population growth

In the presence of *L. niger*,* M. fuscoviride* colonies were more persistent than in the absence of ants, while the differences between no ant and *M. rubra* treatments, and between the *L. niger* and *M. rubra* treatments, were not significant (Table 3, Figure 2b). In the absence of ants, colony persistence was longest in the “no‐competition” treatment (*M. fuscoviride* on its own), while the presence of *M. tanacetaria* or *U. tanaceti* on the plant decreased colony persistence by about 50% and 80%, respectively (Table 2). In the three‐species treatment, colony persistence decreased also by about 60% in comparison with the no‐competition treatment. Competition had no any effect on the colony persistence of *M. fuscoviride* in the presence of ants, *L. niger* or *M. rubra* (Table 2).

**Table 3:** The overall effect of the presence of ants on colony persistence, in days, of three specialized tansy aphids in the greenhouse and field experiments

	Mean ± *SE*	*Lasius niger*	*Myrmica rubra*
		χ^2^	χ^2^
Greenhouse			
*M. tanacetaria*			
Without ants	17.33 ± 0.97	27.38***	30.92***
*Lasius niger*	7.08 ± 1.24		0.004
*Myrmica rubra*	7.38 ± 1.10		
*M. fuscoviride*			
Without ants	14.58 ± 1.19	8.15**	3.13
*Lasius niger*	18.63 ± 0.70		1.47
*Myrmica rubra*	17.73 ± 0.89		
*U. tanaceti*			
Without ants	18.33 ± 0.80	10.4**	8.08**
*Lasius niger*	14.43 ± 1.23		0.17
*Myrmica rubra*	14.95 ± 1.22		
Field			
*M. tanacetaria*			
Without ants	14.21 ± 0.40	100.13***	
*Lasius niger*	7.23 ± 0.40		
*M. fuscoviride*			
Without ants	3.03 ± 0.15	170.47***	
*Lasius niger*	15.35 ± 0.65		
*U. tanaceti*			
Without ants	10.74 ± 0.29	21.33***	
*Lasius niger*	12.73 ± 0.52		

The ant treatment had three levels in the case of the greenhouse experiment (*L. niger*,* M. rubra*, without ant), and two levels in the case of the field experiment (with/without *L. niger*). For the analysis of colony persistence, a survival analysis (Kaplan–Meier) was used. Pairwise comparison between ant treatments was performed using the *log‐rank* test.

Overall mean ± *SE* and chi‐square (χ^2^) are given, and significant results are indicated by **p <* *.01, ***p <* *.001.

Considering the cumulative number of individuals, the interaction between the ant and competition treatments was significant (Table 1). The presence of ants significantly increased the cumulative number of *M. fuscoviride* individuals, resulting in about six and four times more individuals in the presence of *L. niger* and *M. rubra*, respectively, than in the no ant treatment (Figure 1e, Table 1). In the absence of ants, population growth of *M. fuscoviride* was very slow and even lower in the presence of *M. tanacetaria*, while *U. tanaceti* had no considerable effect. In the presence of ants, competition had no negative effect on cumulative number of *M. fuscoviride* individuals (Figure 1e).

#### 3.1.3 *Uroleucon tanaceti* (untended)

##### Early adult survival

In the absence of ants, early adult survival of *U. tanaceti* was more than 1.5 times greater than in the presence of ants (Figure 1c, Table 1). Competition affected early adult survival (Table 1), such that in the presence of ants and *M. tanacetaria,* the early adult survival was higher than other treatments. The interaction between competition and ant treatment was not significant (Table 1).

##### Colony persistence and population growth

Colony persistence of *U. tanaceti* was significantly shorter in the presence of *L. niger* and *M. rubra* than in the absence of ants (Table 3, Figure 2c). There was a tendency for lower colony persistence in competition with *M. fuscoviride* in the presence of *L. niger* (Table 2).

The presence of ants also had a negative effect on the cumulative number of *U. tanaceti* individuals, and the effects of *L. niger* tended to be stronger than that of *M. rubra* (Figure 1f). Competition had no significant effect on the cumulative number of *U. tanaceti* individuals and also the interaction between ant and competition treatment was not significant (Table 1). Patterns of population growth of *U. tanaceti* were, however, complicated (Figure 1f). While populations of *U. tanaceti* grew well in the absence of ants; the cumulative number of *U. tanaceti* individuals in the presence of *M. tanacetaria* or *M. fuscoviride* with *M. rubra* was the same as in the “no ants” treatment (Figure 1f).

### 3.2 Field experiments

#### 3.2.1 Competition experiment

##### *Macrosiphoniella tanacetaria* colony persistence and population growth

The presence of the ant *L. niger* halved colony persistence of *M. tanacetaria* (Figure 2d, Tables 2 and 3). Competition also had a negative effect on *M. tanacetaria* colony persistence in the absence of ants, when it was accompanied by *U. tanaceti* (Table 2). In the presence of ants, competition negatively affected the colony persistence of *M. tanacetaria* such that the persistence time was about half as long as when the aphid was without competitors (Table 2).

**Figure 3:**
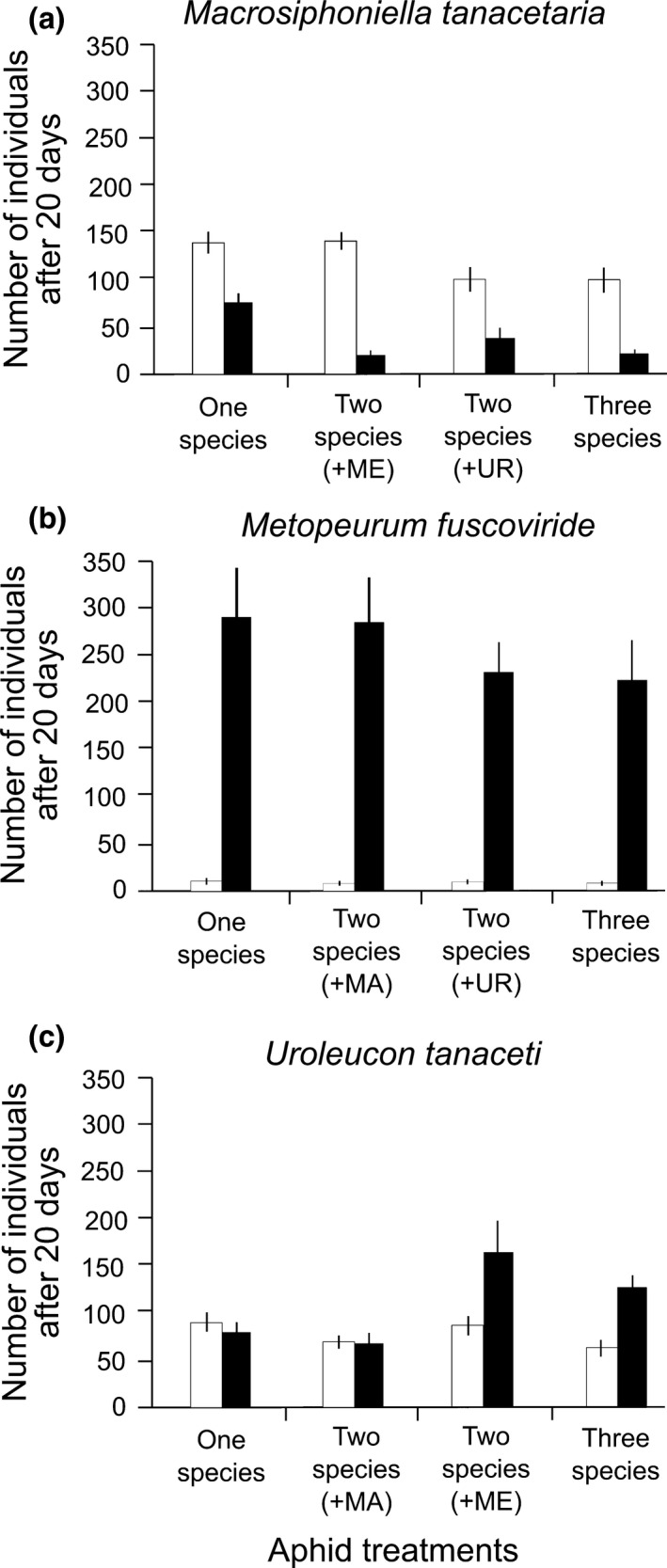
The effect of ant presence and aphid‐aphid competition on population growth (cumulative number of individuals) of specialized tansy aphids in the field experiment. Aphid competition treatments included the focal aphid species on its own, the combination of the focal with either of the two other aphids species, and the three‐species combination. The ant treatment had two levels (with/without *L. niger*). Mean (±*SE*) cumulative numbers of *Macrosiphoniella tanacetaria* (a), *Metopeurum fuscoviride* (b), and *Uroleucon tanaceti* (c) individuals during 21 experimental days in different aphid (competition) treatments in the field experiment are shown. White columns: no ants, black columns: with ants (*Lasius niger*). MA:* M. tanacetaria*; ME:* M. fuscoviride*; UR:* U. tanaceti*

The cumulative number of individuals after 21 days for *M. tanacetaria* was about 3.5 times larger without than with *L. niger* present (Figure 3a). The interaction between ant and competition treatments was significant (Table 1). In the absence of ants, *M. fuscoviride* apparently had no effect on the population growth of *M. tanacetaria*, while the negative effect of *U. tanaceti* was found to be significant. In the presence of ants, competition decreased the cumulative number of individuals in all competition treatments, more especially when *M. fuscoviride* was present, so that it was greater in the “*M. tanacetaria* alone” treatment compared with the other aphid combinations (Figure 3a).

##### *Metopeurum fuscoviride* colony persistence and population growth

As with the greenhouse experiment, the ants had a positive effect on colony persistence of *M. fuscoviride*, and colonies persisted on average about five times as long when they were not attended by ants (Tables 2 and 3). Competition had no discernible effect on the colony persistence of *M. fuscoviride* (Table 2). In the “no ant treatment,” colony persistence was very short independent of the competition treatment, colonies rarely survived beyond the first 3 days.

In the presence of *L. niger*, populations grew considerably and the cumulative number of individuals were high, about 33‐fold the average of the no ant treatment, in all combinations with other aphids or when alone. In contrast, in the absence of ants, colonies became extinct in the first 3 or 4 days due to the action of predators in all aphid species combinations (Table 1, Figures 2e and 3b). The main effect of competition and the interaction between ant and competition were hence not significant (Table 1).

##### *Uroleucon tanaceti* colony persistence and population growth

In contrast to the greenhouse experiment, the presence of ants caused an overall increase in colony persistence of *U. tanaceti* (Table 2, Figure 2f). Competition had no effect on the colony persistence of *U. tanaceti* in the absence of ants, but the presence of *M. fuscoviride* and ants on the plant caused an increase in colony persistence of *U. tanaceti* (Table 2).

With regard to the cumulative number of *U. tanaceti* individuals, the interaction between ant and competition was marginally significant, and patterns were complicated (Table 1, Figure 3c). In the absence of ants, there was no effect of competition on the cumulative number of *U. tanaceti* individuals, while the presence of ants generally increased the number, in comparison with no ant treatments. *Uroleucon tanaceti* had the highest growth rate in the presence of *M. fuscoviride* and with access of ants, while it was worse in the presence of *M. tanacetaria*, independent of ant presence (Figure 3c).

#### 3.2.2 Colonization experiment

**Figure 4:**
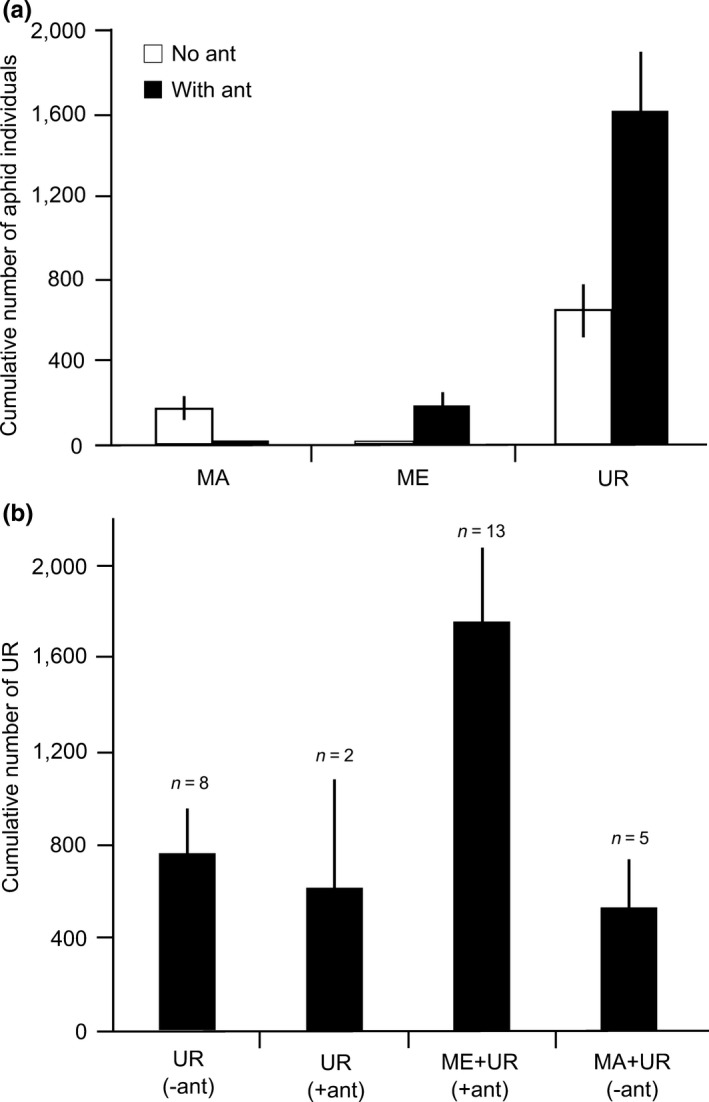
Cumulative number of aphid individuals during the experiment in “no ant” and “ant” treatments (a), cumulative number of *Uroleucon tanaceti* in different combination with other aphids during the entire period of the experiment (b). MA:* M. tanacetaria*; ME:* M. fuscoviride*; UR:* U. tanaceti*

Assembly dynamics depended on the presence of ants. *Macrosiphoniella tanacetaria* were observed colonizing and establishing a colony only on plants where ants were absent while *M. fuscoviride* did so where the ants are present (Table 4, Figure 4a). *Uroleucon tanaceti* mostly colonized plants with ants; however, plants without ants were also colonized (Table 4, Figure 4a). The cumulative number of *U. tanaceti* in combination with other species was higher in ant treatments where *M. fuscoviride* also cooccurred (Figure 4b). In figure 4, *U. tanaceti* is the only aphid included because (i) it was the most abundant species on most of the plants, (ii) it colonized the plants with and without ants, and (iii) it showed the most complicated patterns. In the first 3 weeks of the experiment, 13 of the 15 plants with access of ants were colonized by both *M. fuscoviride* and *U. tanaceti*. The cooccurrence of *M. tanacetaria* and *M. fuscoviride* was never observed during the experiment; however, the cooccurrence of *M. tanacetaria* with *U. tanaceti* was observed only in no ant treatments. *Metopeurum fuscoviride* cooccurred with *U. tanaceti* on plants where the ants were present (Table 4).

**Table 4:** Aphid colonization and occupancy in the field colonization experiment

	Unoccupied	MA	ME	UR	MA + ME	MA + UR	ME + UR	MA + ME + UR
No. of colonizations (in the first 3 weeks)								
+ ant (*N* = 15)	0	0	0	2	0	0	13	0
− ant (*N* = 15)	0	0	0	8	0	5	1	1
Dominant aphid species (over the entire period)								
+ ant (*N* = 15)	0	0	0	15				
− ant (*N* = 15)	0	2	0	13				
Established colonies (at least 1 week) over the entire period								
+ ant (*N* = 39)	0	0	10	29				
− ant (*N* = 34)	0	15	0	19				
Established colonies (at least 1 week) cooccurring at the same time								
+ ant (*N* = 29)	0	0	4^x^	15	0	0	10^x^	0
− ant (*N* = 29)	0	9	0	13	0	7	0	0

4^x^ means that four plants had a period where there was only a ME colony on the plant, 10^x^ means that 10 plants had a period when both ME and UR colonies were on the plant. The same plant may occur more often in the table, for example, when first there was an established ME colony, and then there were simultaneously ME and UR colonies. Shaded area means these comparisons are not applicable. MA: *M. tanacetaria*; ME: *M. fuscoviride*; UR: *U. tanaceti*.

**Figure 5:**
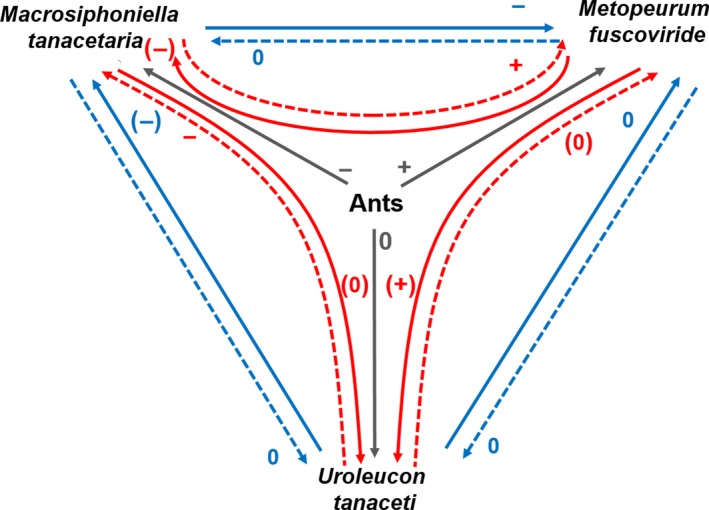
Interaction web of tansy aphids including indirect effect of ants. Blue arrows show interactions between two aphid species in the absence of ants, red arrows show interactions in the presence of ants. Gray arrows show main effect of ants on the different aphid species. + positive effect; − negative effect, 0 neutral effect. Effects in brackets are tendencies, for example, effect only significant in field experiment but not in greenhouse experiment (see Table 2). Dashed and solid lines highlight the direction of the arrow, from one aphid to the other (solid) and vice versa (dashed)

## 4 DISCUSSION

Our results show that the presence of ants drives community assembly of the three specialized tansy aphids at the local level (individual plants), by affecting aphid–aphid competition through mutualistic or predatory interactions with different species (Figure 5). For the ant‐tended *M. fuscoviride,* the interactions with ants are mutualistic, resulting in competitive dominance in the presence of ants, and competitive exclusion in the absence of ants. The untended *M. tanacetaria* is competitively superior in the absence of ants, but the interaction with ants is characterized by ant predation of aphids, resulting in local extinction. For the untended *U. tanaceti,* the net effect of ant presence is positive only in the presence of *M. fuscoviride* and other predators, because the presence of *M. fuscoviride* consequently attracted ants and in result, all aphids were protected against natural enemies. Therefore, the presence of ant‐tended *M. fuscoviride* aphids had a strong *indirect* negative effect on *M. tanacetaria* aphids in the presence of ants on the plant, because ants removed *M. tanacetaria* around the *M. fuscoviride* colony.

The greenhouse results were consistent with observed patterns in the field colonization experiment, where ant presence determined which aphid was able to colonize the plant successfully. Thus, our results clearly show that coexistence at the local level does not occur in these three aphid species. Instead, the presence of other organisms, in particular ants and (other) insect predators such as ladybirds or lacewings, determines which aphid species persists on a particular plant. Because ants affect the competitive hierarchy among aphid species, coexistence at the regional level is possible when there is variation in ant presence across individual plants. While ants are potentially present everywhere, they may not necessarily visit every plant to the same degree. Recent work indicates that ants show preferences due to plant chemical variation (Clancy, Zytynska, Senft, Weisser, & Schnitzler, 2016; Senft et al., 2017). Therefore, we must begin to consider this system at the regional level, using our understanding of the local community dynamics in order to understand fully what drives species interactions and coexistence of the different aphid species.

### 4.1 Local interactions among aphids and the role of ants

The ant‐tended *M. fuscoviride* benefits from ants in different ways, for example, protection against natural enemies (Billick et al., 2007; Tilles & Wood, 1982; Way, 1963). This can indirectly benefit other species, for example, *U. tanaceti* aphids on the same host plant assuming they are ignored by the ants directly. Here, we also show that the ants benefit *M. fuscoviride* aphids by eliminating the competitors as soon as they arrived on the plant (early adult survival in our experiments). Our results suggest this protection is species‐specific, with ants predating *M. tanacetaria* (Figure S1) but not *U. tanaceti*. Without ants, *M. fuscoviride* is competitively inferior to *M. tanacetaria*. The extinction of *M. fuscoviride* aphids on plants without ants is likely driven by a combination of various mechanisms. For example, predation by insect predators, poor survival, and reproduction when ants do not remove honeydew from individuals (Benedek et al., 2015; Flatt & Weisser, 2000; Mehrparvar, Zytynska et al., 2014; Mehrparvar et al., 2013), and possibly competition for plant resources with other aphids, in particular *M. tanacetaria* (as there was no effect of *U. tanaceti* on *M. fuscoviride*).

The strong negative effect of ants on *M. tanacetaria* colony persistence and population growth was consistent across all combinations of aphid species. This occurred in two main ways: Firstly, ants preyed on *M. tanacetaria* and secondly, the ants disturbed *M. tanacetaria* by walking through the colony, which often makes them fall off the host plant and many fail to return and subsequently die. Ants may change from mutualist to predator due to a lower production of honeydew by *M. tanacetaria* and also lower honeydew quality (Woodring, Wiedemann, Fischer, Hoffmann, & Völkl, 2004), so that there is little return to the ants when tending this aphid species (see Fischer, Hoffmann, & Völkl, 2001; Sakata, 1994, 1995; Skinner, 1980). Even though *U. tanaceti* belongs to the same group of herbivores on the tansy plant, there is nevertheless a different and somehow complicated impact of ants on it. Ants occasionally visited the *U. tanaceti* colonies on the plant, but both attendance and predation were rare. It is known that *U. tanaceti* can be toxic to some common aphid predators (Mehrparvar, Mahdavi Arab et al., 2013) and perhaps also to the ants, thus explaining the ant avoidance of this aphid species.

The case of *U. tanaceti* is interesting as the fitness of *M. tanacetaria* (in terms of cumulative number and colony persistence) was reduced in the presence of *U. tanaceti*. While *M. tanacetaria* lives on the top of the plant while *U. tanaceti* lives on the lower parts, the underside of the leaves. Competitive interactions can occur both directly and indirectly (Begon, Townsend, & Harper, 2006). In the case of two aphid species, even if each contending species occupies a different part of the plant, there may still be indirect competition for phloem nutrients, in particular amino acids. As shown by Moran and Whitham (1990), even two aphid species that feed on two different parts of a plant, root, and leaf, can affect each other via competitive interactions mediated by the host plant. This may also be the case of the untended *U. tanaceti* when present on the same plant as *M. tanacetaria*. These competitive interactions were asymmetric, as *Macrosiphoniella tanacetaria* had a negative effect on the population growth of *U. tanaceti*, but not on colony persistence. As populations of *U. tanaceti* and *M. tanacetaria* in the field experiment did not grow much but were instead kept at low density due to predation, such competition had little discernible effect on population growth.

In the presence of ants, there was no observed negative effect of *U. tanaceti* on *M. fuscoviride* colony persistence, but the population growth of *M. fuscoviride* was smaller. Apparently, ants had little effect on *U. tanaceti*. Yet, the presence of *M. fuscoviride* on the plants in the field, which consequently attracted ants, had a clear *positive* effect on population growth and colony persistence of *U. tanaceti*. The reason for this is seemingly that natural enemies cannot hold sway on the plant because of the ants, which are attending *M. fuscoviride*, but not *U. tanaceti*. This in effect results in an enemy‐free environment for *U. tanaceti*, enhancing fitness and allowing for coexistence with *M. fuscoviride*. Nevertheless, if the population of both or one of the species increases to high numbers, it could definitely lead to competition (Denno, Mcclure, & Ott, 1995).

### 4.2 Coexistence across host plants

Overall, our experiments show that bilateral interactions between the three aphid species make it very unlikely that all three species coexist at the level of an individual plant. Instead, community assembly depends on the presence of ants and of insect predators and results in one or two‐species communities. In the field, colonies of *M. fuscoviride* and *M. tanacetaria* are rarely found coexisting on the same plant (Loxdale et al., 2011). We suggest that the main driver of community assembly in tansy is the mutualistic interaction between ants and *M. fuscoviride*. The negative effect of ants on *M. tanacetaria* was strongest on initial colonization of a patch, never allowing these aphids to build up a high population size. The positive effect of ants on *M. fuscoviride* was to enable high population growth after initial colonization, leading to high colony persistence, and reduced chance of extinction. As a result, the change in competitive hierarchy due to the presence of ants, coexistence between the three species of aphids, and in particular between *M. fuscoviride* and *M. tanacetaria* is only possible across multiple host plants in a field. Without the competitive superiority of *M. tanacetaria* in the absence of ants, it is unlikely that these species would maintain the coexistence pattern observed. The outcome of the community assembly process is, in fact, predictable once the presence of ants and (other) aphid predators are known, as shown in our field colonization experiment. In this experiment, we determined whether a plant was tended by ants or not, thereby affecting the community assembly process on each experimental plant. How would such a process work under more natural conditions, that is, when ants are free to choose what plants to colonize?

Aphid‐tending ants are ubiquitous in terrestrial systems and are only absent when disturbances do not allow sufficient time to develop a colony (e.g., in arable land) or when abiotic conditions are too harsh, in particular when soils are wet or frequently flooded. Along rivers and on islands, for example, near the Finnish coast (Weisser & Harri, 2005), tansy occurs in well‐drained sites that are rarely flooded; the wastelands it colonizes are also generally dry, and ant colonies can be found in the vicinity of tansy plants. Thus, even in the historic landscape, the absence of ants is unlikely to explain regional coexistence of tansy aphids, in particular the persistence of *M. tanacetaria*. Recent work indicates colonization of plants, and the activity of ants on the plants can be mediated by chemical variation in the plant (Clancy et al., 2016). Thus, intraspecific plant variability may affect aphid population growth not only directly, by changing plant suitability for the aphids, as is well‐known from plant resistance research and also found in natural systems, but also indirect via ant preference (Zytynska & Weisser, 2016). In fact, variation in tansy chemotypes has been found at the level of a field (Clancy et al., 2016). This chemical variation thus creates a heterogeneous habitat for the aphids and hence community assembly processes. Further work is necessary to understand the role plant variation in driving community assembly processes of aphid communities through effects on mutualistic ants and possibly predators. The mechanisms may in fact be even more complicated as colony persistence time in the field (how long a plant is colonized for) is also ant species related, with persistence time being reduced when colonies were tended by *M. rubra* rather than *L. niger* (Senft et al., 2017). In other systems, host plant genetic variation was also able to structure aphid populations indirectly through interactions with mutualistic ants (Abdala‐Roberts, Agrawal, & Mooney, 2012; Mooney & Agrawal, 2008; Wimp & Whitham, 2001).

### 4.3 Conclusions

In the current study, we have experimentally shown that ant mutualistic/antagonistic relationships drive competitive exclusion between two aphid species within local communities (i.e., individual plants), with a third aphid less affected by these interactions. In such a system, with multiple local patches linked by dispersal, we also suggest that understanding how biotic interactions drive community assembly at the local scale can benefit future work in the field of metacommunity ecology, where the effect of these local interactions can be further investigated across multiple patches at the field or regional scale.

## CONFLICT OF INTEREST

None declared.

## AUTHOR CONTRIBUTIONS

MM and WWW conceived and designed the experiments. MM and AB performed the experiments. MM analyzed the data. MM and SEZ wrote the first draft of the manuscript. All authors contributed substantially to revisions.

## DATA ACCESSIBILITY

Data is available from the Dryad Digital Repository https://doi.org/10.5061/dryad.7j2c1.
