# Supplementary figures and images for: Coexistence through mutualist‐dependent reversal of competitive hierarchies
Source: Ecol Evol. 2017 Dec 21;8(2):1247–59. doi: 10.1002/ece3.3689 (PMC5773332; doi:10.1002/ece3.3689)

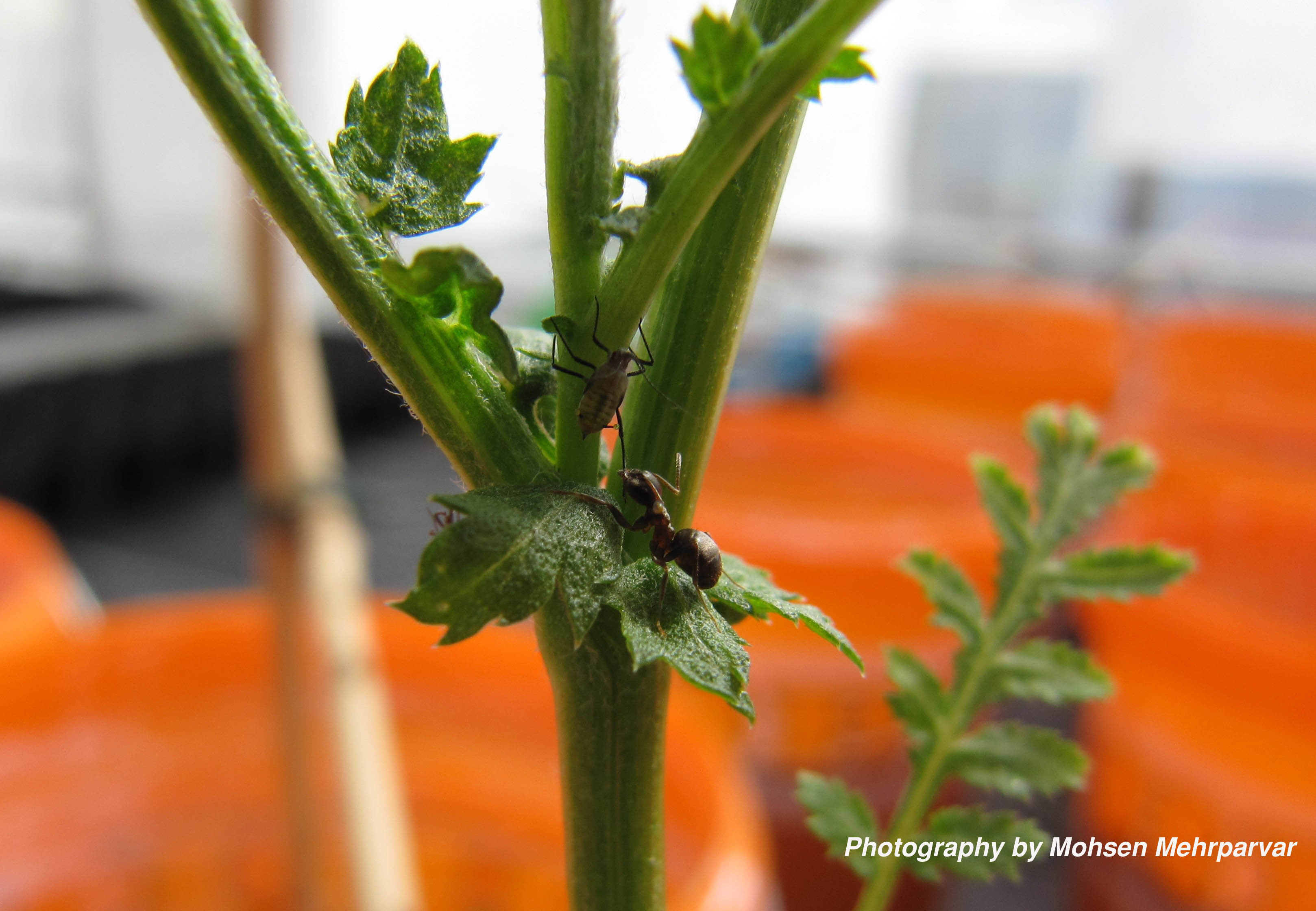

Supplement: Supplementary file 1 [file ECE3-8-1247-s001.tiff]
